# First European Identification of a Partial Tacheng Tick Virus 8-like Underscores the Hidden Burden of Tick-Borne Flavivirus-like Viruses

**DOI:** 10.3390/v18090984

**Published:** 2026-09-08

**Authors:** Guilherme Moreira, Eliane Silva, Joana Mourão, João R. Mesquita

**Affiliations:** 1 School of Medicine and Biomedical Sciences (ICBAS), Universidade do Porto, 4050-313 Porto, Portugal; grmoreira@icbas.up.pt (G.M.); elianepimenta05@gmail.com (E.S.); 2 Research Center in Biodiversity and Genetic Resources (CIBIO/InBIO), University of Porto, Rua Padre Amando Quintas, nr. 7, 4485-661 Vairão, Portugal; 3 BIOPOLIS Program in Genomics, Biodiversity and Land Planning, Research Centre in Biodiversity and Genetic Resources (CIBIO), Campus de Vairão, 4485-661 Vairão, Portugal; 4 National Food Institute, Technical University of Denmark, Kongens Lyngby, 2800 Copenhagen, Denmark; joam@food.dtu.dk; 5 Laboratório Associado para a Química Verde-Tecnologias e Processos Limpos (LAQV-REQUIMTE), Praça Coronel Pacheco, n° 15-6° Andar, 4050-453 Porto, Portugal

**Keywords:** Tacheng tick virus 8, flavivirus, metagenomics, tick-borne viruses, virome, Portugal

## Abstract

Tick-associated viruses are an underexplored component of global viromes, and highly divergent RNA viruses often remain undetected due to low abundance and sequence divergence. Tacheng tick virus 8 (TcTV8) is a poorly characterized RNA flavivirus-like virus originally reported from Dermacentor-associated ticks in China, with few subsequent reports and no confirmed detections outside Asia. Here, we report the detection and partial genomic characterization of a TcTV8-like virus in ticks collected from Portugal. Sequence-independent (SISPA) nanopore sequencing of individual ticks recovered three partial fragments (~2.7 kb, ~14% of the reference genome; ~30% of the reference covered at ≥1×), which shared high amino-acid identity with the TcTV8 polyprotein, including a methyltransferase-region domain. Phylogenetic analysis placed the Portuguese sequence within the TcTV8 lineage. Read-level classification and coverage analysis further support the presence of this virus in the positive sample. These findings represent, to our knowledge, the first detection of a TcTV8-like virus in European ticks, extending its known geographic range beyond Asia. The detection of this cryptic viral lineage highlights the need for broader tick virome surveillance to better understand the diversity, evolution, and ecology of flavivirus-like viruses.

## 1. Introduction

Ticks are among the most important arthropod vectors of infectious diseases and are increasingly recognized as reservoirs of an extraordinarily diverse and highly divergent assemblage of RNA viruses [1,2]. Advances in metagenomic and metatranscriptomic sequencing have revealed that ticks from a wide range of species and geographic regions harbor dozens of viral lineages, spanning multiple viral families and encompassing a broad array of genome architectures, including positive-, negative-, and double-stranded RNA viruses, as well as segmented and non-segmented genomes [1,3]. While some of these viruses appear to be tick-specific and reflect long-term virus–arthropod coevolution, others belong to broader evolutionary lineages that include established human and veterinary pathogens, such as tick-borne encephalitis virus, severe fever with thrombocytopenia syndrome virus, and Nairobi sheep disease virus [1,4]. Despite these rapidly expanding virome datasets, the host range, transmission potential, and pathogenic relevance of most tick-associated viruses remain poorly understood, underscoring the need for systematic genomic and ecological investigations to assess their significance for public and animal health [1,5,6].

Among the tick-associated viruses of greatest medical and veterinary concern are members of the family Flaviviridae. According to the International Committee on Taxonomy of Viruses (ICTV), the family Flaviviridae (order Amarillovirales) currently comprises five genera, including the genus Orthoflavivirus (formerly Flavivirus). A major 2025 reclassification further incorporated numerous divergent flavi-like viruses into the family, including the segmented Jingmenvirus genus and other large-genome flavi-like lineages, spanning mosquito-borne, tick-borne, and insect-specific viruses, as well as viruses without a known arthropod vector [7]. Members of this group are globally distributed and include numerous pathogens of major medical and veterinary importance. Prominent mosquito-borne examples include dengue, West Nile, Japanese encephalitis, yellow fever, and Zika viruses, while tick-borne encephalitis virus represents one of the most significant tick-associated members, together accounting for hundreds of millions of infections annually [8]. Most flaviviruses cycle between arthropod vectors and vertebrate hosts, although insect-specific and apparently vector-independent lineages have also been described [7].

Tacheng tick virus 8 (TcTV8) is a positive-sense RNA flavi-like virus with a large (~19.5 kb) genome, first described through metagenomic sequencing of Dermacentor-associated ticks in Tacheng, northwestern China [9]. Despite the shared “Tacheng tick virus” designation, TcTV8 is not closely related phylogenetically to Tacheng tick viruses 1 and 2. TcTV-8 is an unclassified positive-sense ssRNA flavi-like virus, whereas TcTV-1 and TcTV-2 are negative-sense RNA viruses belonging to the families Nairoviridae and Phenuiviridae, respectively. Like other flavi-like viruses, TcTV8 is presumed to encode a single large polyprotein bearing the conserved methyltransferase and RNA-dependent RNA polymerase (NS5-like) motifs characteristic of the group, although its full genome organization, structural protein complement, and vector or vertebrate host range remain unresolved [10,11]. A partial NS5-coding region of TcTV8 has since been reported in ticks from Anatolia, Turkey, and a further possible detection has been described from Kenya. These reports, however, describe the sequences only within the original publications [9,12,13]. Crucially, the corresponding sequence data were not deposited in public repositories, so these detections remain documented only as reports and cannot be independently verified or compared at the genomic level. Taken together, these reports suggest that TcTV8-like viruses may be geographically more widespread than the single well-characterized Chinese genome would indicate. This broader distribution nonetheless remains unconfirmed, as the non-Chinese detections are limited to short, partial regions without deposited sequences.

Despite this apparent breadth, TcTV8 itself has not previously been reported in Europe, although other flavi-like viruses (e.g., Jingmen tick virus and Alongshan virus) are already known from European ticks [14,15]. This is unlikely to reflect a genuine biogeographic absence and more plausibly reflects a sampling and surveillance gap. Indeed, unbiased metagenomic and metatranscriptomic tick virome discovery efforts have so far been concentrated disproportionately in Asia, where most novel tick-associated RNA viruses, including TcTV8, have been characterized [1,2,3]. By comparison, systematic, sequence-independent virus discovery in European ticks remains comparatively limited, with most regional tick-pathogen studies relying on targeted PCR assays designed to detect previously characterized agents (e.g., Borrelia, Anaplasma, tick-borne encephalitis virus) [16,17,18]. Unfortunately, this approach is inherently blind to divergent or previously undescribed viral lineages such as TcTV8. Consequently, the true diversity and distribution of flavivirus-like viruses in European tick populations remains largely unknown. Lineages such as TcTV8 could plausibly have gone undetected due to a combination of low viral loads in individual ticks, high sequence divergence limiting the sensitivity of established diagnostic assays, and the comparative scarcity of unbiased sequencing-based surveillance efforts in different regions.

The Algarve region of southern Portugal is an area characterized by a Mediterranean climate, high biodiversity, and extensive agro-pastoral activity, where livestock are commonly raised under free-ranging or semi-extensive systems near human settlements [19]. The region also represents an important ecological interface for migratory birds, wildlife, and domestic animals, creating favorable conditions for the maintenance and exchange of tick populations and their associated pathogens [20,21]. Ongoing climate change, including rising temperatures and altered precipitation patterns, is expected to further expand tick habitats, prolong seasonal activity, and facilitate the northward and altitudinal spread of vector species in southern Europe [22]. These environmental and socio-ecological factors increase the likelihood of pathogen introduction, amplification, and spillover between wildlife, livestock, and humans. These interactions thereby heighten the risk of emergence of previously unrecognized tick-borne viruses and justify the need for unbiased metagenomic approaches for the assessment of highly divergent viral circulation in arthropod vectors.

The primary aim of this study was to screen ticks collected in Portugal for divergent, previously undescribed viruses using an unbiased metagenomic approach. Here, we report the detection and preliminary genomic characterization of a TcTV8-like virus from these ticks, based on partial genome recovery. This detection extends the known geographic range of TcTV8-like viruses into Europe and provides new insight into the phylogenetic relationships of these widely distributed, cryptic tick-associated lineages, underscoring the value of comprehensive tick virome surveillance.

## 2. Materials and Methods

### 2.1. Sample Collection and RNA Extraction

A total of 10 ticks were collected from multiple host species between July 2024 and Jan 2025. All samples originated from the Algarve region of Portugal. Ticks were removed manually using sterile fine forceps during routine veterinary procedures and transported alive to the laboratory under humidified conditions following standard handling protocols for arthropods intended for downstream molecular analyses [23]. These ticks represented a subset from a larger collection, selected to encompass diverse host species and tick genera. Ticks were morphologically identified to the genus level using standard taxonomic keys under a stereomicroscope SMZ800N (Nikon, Tokyo, Japan), following the methodology described by Estrada-Peña et al. [24]. Observations focused on key diagnostic features such as capitulum structure, scutum ornamentation, and leg segmentation to ensure accurate identification. Details regarding collection dates and host species are summarized in Table 1.

Total RNA was extracted from whole single ticks using the QIAamp Viral RNA Mini Kit (Qiagen, Hilden, Germany) and the QIAcube^®^ automated platform (Qiagen, Hilden, Germany), with protocol modifications to include mechanical homogenization and proteinase K digestion. Prior to RNA extraction, ticks were superficially sterilized by sequential immersion in 70% ethanol, followed by sterile nuclease-free water, and allowed to air dry under sterile conditions. The tick samples were then homogenized using a TissueLyser bead mill (Qiagen, Hilden, Germany) in the presence of approximately 400 μL of AVL buffer (Qiagen, Hilden, Germany) and 25 μL of proteinase K. They were then incubated at 37 °C for 10 min to facilitate lysis and protein digestion. After incubation, AVL buffer was added to bring the final volume to 700 μL, followed by brief centrifugation to remove debris. The clarified lysate was then transferred to the QIAcube instrument, and total RNA was extracted using the Viral RNA Mini Kit protocol with off-board lysis. RNA was eluted in 60 μL of RNase-free water and stored at −80 °C until further use.

### 2.2. Sequence Independent Single Primer Amplification (SISPA)

Following RNA extraction, residual genomic DNA was removed by treatment with RNase-free DNase I (Qiagen, Hilden, Germany) in accordance with the manufacturer’s protocol. The DNase-treated RNA served as the template for sequence-independent single-primer amplification (SISPA) [25], enabling non-biased reverse transcription and subsequent amplification of RNA-derived templates. First-strand complementary DNA (cDNA) synthesis was carried out using SuperScript IV reverse transcriptase (Thermo Fisher Scientific, Waltham, MA, USA) in conjunction with a primer consisting of a defined 5′ anchor sequence linked to a random nonamer (5′-GTTTCCCACTGGAGGATA-N9-3′). Reactions were incubated at 25 °C for 10 min, followed by 50 °C for 50 min, and enzyme inactivation at 70 °C for 15 min. Second-strand cDNA synthesis was performed using Sequenase Version 2.0 DNA Polymerase (Thermo Fisher Scientific) via a two-step extension protocol. Sequenase was first added directly to the first-strand reaction mixture and incubated at 37 °C for 10 min, after which a second aliquot of Sequenase was introduced and the reaction was incubated for an additional 10 min at 37 °C to ensure complete synthesis of the complementary strand. No purification steps were performed between the first- and second-strand synthesis reactions. The resulting double-stranded cDNA was subsequently amplified by polymerase chain reaction (PCR) using a primer corresponding to the defined anchor sequence (5′-GTTTCCCACTGGAGGATA-3′) and Q5 High-Fidelity DNA Polymerase (New England Biolabs, Ipswich, MA, USA). Thermal cycling was conducted as follows: initial denaturation at 98 °C for 30 s; 35 amplification cycles of 98 °C for 10 s, 55 °C for 20 s, and 72 °C for 30 s; followed by a final extension at 72 °C for 2 min. Final concentrations were normalized to 15 ng/uL.

### 2.3. Oxford Nanopore Sequencing

SISPA-prepared cDNA was sequenced using Oxford Nanopore Technology (Oxford Nanopore Technologies (ONT), Oxford, UK) via a PromethION 24 instrument (Oxford Nanopore Technologies (ONT), Oxford, UK), equipped with an R10.4.1 flow cell. The library preparation utilized the Native Barcoding Kit 96 V14 (SQK-NBD114.96) (Oxford Nanopore Technologies (ONT), Oxford, UK), following the manufacturer’s instructions. The raw FASTQ reads were basecalled in super-accurate mode, using the Dorado basecaller (ont-doradod-for-promethion) v7.4.12, applying a minimum Q-score of 10, with adapters and barcodes trimmed via MinKNOW v6.0.4. Initial quality control of the sequencing reads was performed using NanoPlot 1.43.0 and sequencing adapters and barcodes were removed using Porechop 0.2.4. NanoFilt v.2.8.0 was employed to filter lower-quality reads, applying a minimum average quality score threshold of 10 and a minimum length of 100 bp. The resulting high-quality reads were used for downstream analyses.

### 2.4. Taxonomic Classification

Taxonomic classification of filtered reads was performed using Kraken2 (v2.17.1) [26]. Classification was carried out using a publicly available Kraken2 database (PlusPFP, version 10/15/2025) built from the NCBI RefSeq collection, including complete and representative genomes of bacteria, archaea, viruses, fungi, plants, and protozoa [26]. Kraken2 was run with default parameters for k-mer length, minimizer length, and minimizer spacing, and a confidence score of 0.1 (--confidence 0.1) was applied. All reads were assigned to the lowest common ancestor (LCA) of the matched reference sequences.

### 2.5. Read Mapping, Coverage Analysis, and Viral Sequence Recovery

Reads taxonomically classified as viral/TcTV8-like were aligned against the TcTV8 reference genome (GenBank accession KR902741; RefSeq accession NC_028367) using Minimap2 v2.30 with the -ax map-ont preset [27]. Alignment files were converted from SAM to BAM format, sorted, and indexed using Samtools v1.22 [28].

Per-base sequencing depth was calculated from the sorted BAM files using Pysam v0.23.3 by extracting per-base coverage from pileup columns. Mean sequencing depth across the complete reference genome was calculated including positions with zero coverage, whereas mean depth across covered positions was calculated considering only positions with ≥1 mapped read. Genome breadth of coverage was calculated as the proportion of reference positions covered by at least one read. Coverage profiles were visualized using Matplotlib v3.10, with coverage calculated in 50 bp sliding windows across regions covered by at least one read. Reads mapping to TcTV8 were subsequently assembled de novo using Flye v2.9.1 [29] to generate contigs. The resulting reads and contigs were then mapped against the TcTV8 reference genome using RagTag [30], and the recovered TcTV8-like coding fragments were used for downstream analyses.

### 2.6. Protein Sequence Annotation and Homology Analysis

The recovered TcTV8-like nucleotide fragments, corresponding to partial regions of the viral polyprotein, were translated in the reading frame corresponding to the TcTV8 reference polyprotein. The resulting partial protein sequences were subjected to functional annotation using InterProScan v5.59-91.0 [31]. Additional profile-based homology analyses were performed using HHpred and HHblits through the MPI Bioinformatics toolkit [32], accessed on the 20 December 2025. Searches were performed against UniRef30_2023_02 (HHblits) and PDB_mmCIF70_20_Feb, Pfam-A_v38.2 and UniProt-SwissProt-viral70_3_Nov_2021 (HHpred) using default parameters unless otherwise stated. The methyltransferase-like region identified within Fragment_3 was further analyzed using HHpred to investigate remote protein homology.

### 2.7. Phylogenetic Analysis

Phylogenetic analysis was performed to determine the taxonomic placement of the detected virus within flaviviruses and flavivirus-like viruses. The three recovered partial genomic fragments were used as query sequences. For each fragment, the corresponding homologous coding region was identified in reference sequences representing recognized members of these groups (GenBank accession KR902741; RefSeq accession NC_028367) by BLASTn (NCBI, accessed September 2025). Only the homologous regions shared between the Portuguese sequences and the reference taxa were retained for downstream analysis. These regions were aligned at the nucleotide level using MAFFT v7.525 [33] and subsequently concatenated for phylogenetic inference. No codon-aware alignment step was implemented. The phylogenetic tree was inferred using a maximum likelihood approach implemented in IQ-TREE v3.0.1 [34], with the best-fit nucleotide substitution model selected using ModelFinder according to the Bayesian Information Criterion (TIM3 + F + R3). Branch support was assessed using 1000 ultrafast bootstrap replicates. The resulting tree was used to evaluate the phylogenetic placement of the Portuguese TcTV8-like sequence relative to TcTV8 and related viruses.

## 3. Results

### 3.1. Tick Identification and Sequencing Output

#### 3.1.1. Morphological Identification

Ticks were morphologically identified to the genus level using standard taxonomic keys under a stereomicroscope, following the methodology described by Estrada-Peña et al. [24]. Observations focused on key diagnostic features such as capitulum structure, scutum ornamentation, and leg segmentation to ensure accurate identification. The identified genera included *Rhipicephalus*, *Hyalomma*, *Dermacentor*, and *Ixodes*, which are commonly associated with domestic animals and are known vectors of various pathogens (Table 2). 

#### 3.1.2. Quality of the Sequencing Data

Oxford Nanopore sequencing of the SISPA-amplified cDNA generated a total of 55,000 reads across 10 samples, 54,797 of which were retained after quality filtering. The quality assessment report generated by NanoPlot can be found in Table 3.

### 3.2. Viral Read Detection and Mapping to the TcTV8 Reference

#### 3.2.1. Detection of TcTV8-like Reads in a Tick

Only sample 1 of the ten yielded a substantial number of viral reads that could be confidently classified. In the remaining samples, viral-associated reads were rare (no more than two reads per sample) and mostly short (under 200 bp), precluding reliable taxonomic assignment. Sample 1 contained a total of 9972 quality-filtered reads. Kraken2 classification left 1304 reads unclassified, while the remaining reads were predominantly assigned to bacteria and eukaryotes, particularly Ixodidae and *Bos taurus*. Of the 9972 reads, 26 were classified as viral, and all 26 were assigned to Tacheng tick virus 8 (TcTV8) by Kraken2. Sample 1 was therefore selected for subsequent reference-mapping and sequence-recovery analyses.

#### 3.2.2. Reference Mapping and Coverage Analysis

**Figure 1:**
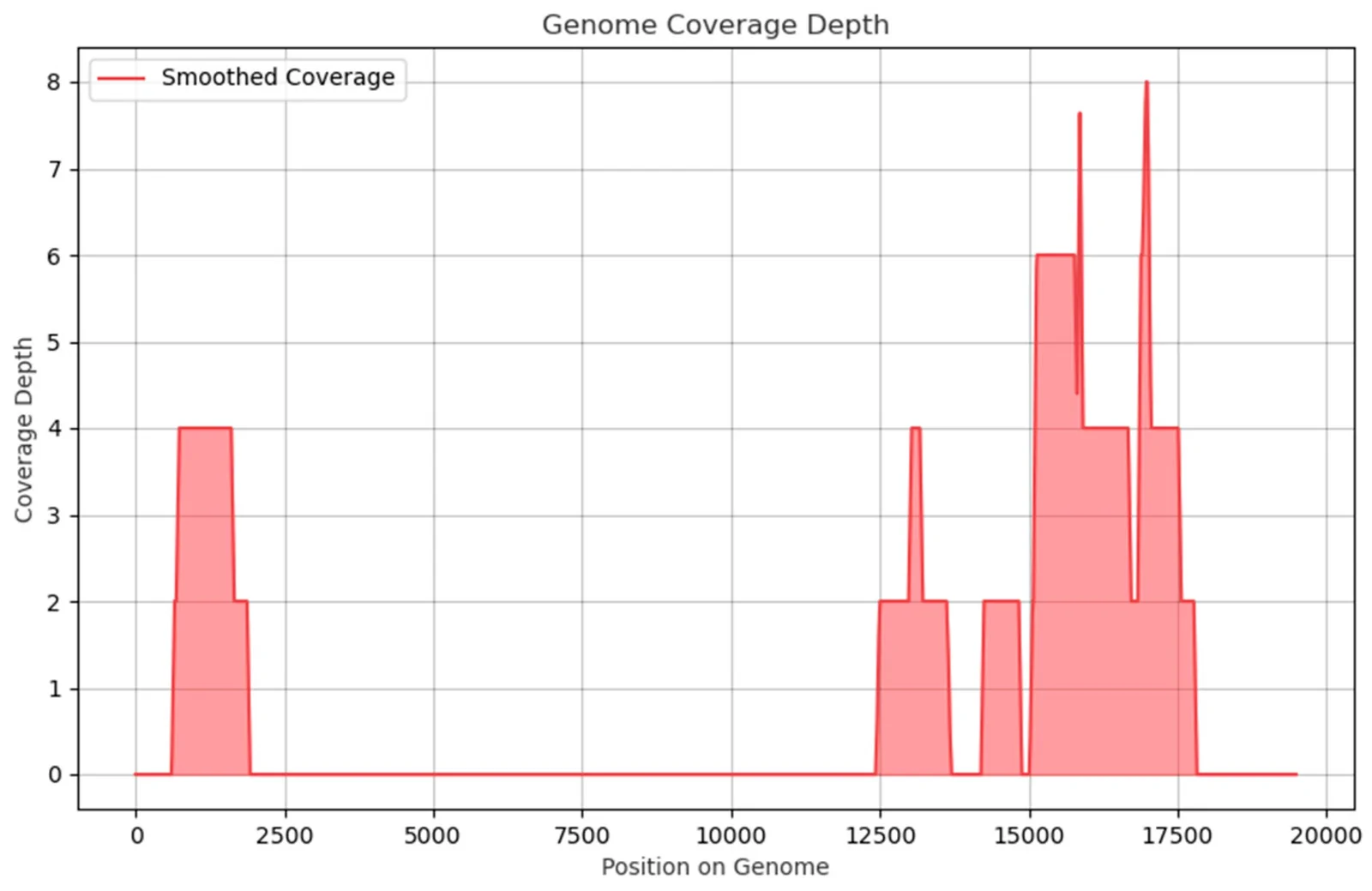
Read-depth coverage across the Tacheng tick virus 8 (TcTV8) reference genome (GenBank accession KR902741; RefSeq accession NC_028367; 19,537 bp) for Sample 1, the only sample positive for TcTV8. Sequencing reads were mapped against the reference genome, and per-base read depth was calculated across the full reference sequence. The *x*-axis represents nucleotide position along the reference genome, and the *y*-axis represents sequencing depth (number of mapped reads). The red trace shows coverage after smoothing using a 50 bp sliding window.

Of the 9972 quality-filtered reads from Sample 1, 26 mapped to the TcTV8 reference genome (GenBank KR902741/RefSeq NC_028367; 19,537 bp), corresponding to 0.26% of the reads from this sample and 0.05% of total sequencing output. The alignment was used to calculate genome coverage and sequencing depth. The mean sequencing depth across the complete reference genome was 1.05×, and the mean depth across positions with ≥1 mapped read was 3.48×. The maximum depth observed at any position was 8×, and 30.18% of the reference genome was covered at ≥1× depth. The coverage profile was visualized as a plot for downstream analysis, as seen in Figure 1. 

### 3.3. Sequence Similarity and Domain Annotation

#### 3.3.1. Recovery of Partial Coding Fragments

Partial fragments were defined by the consensus sequence obtained from the alignment with a TTV8 reference genome. The sequence reconstructed comprised three non-contiguous fragments totaling 2682 bp, corresponding to 13.7% of the reference genome (Table 4). As TcTV8 encodes a single polyprotein, the fragmented nature of the recovered sequence prevented reconstruction of the complete polyprotein.

#### 3.3.2. Protein Feature and Domain Analysis

InterProScan analysis of the translated recovered fragments identified limited protein-feature and domain/superfamily matches. No significant domain matches were detected for Fragment_1 or Fragment_2. Fragment_3showed a significant Gene3D match to the Vaccinia virus VP39-like domain/superfamily (G3DSA:3.40.50.150; E = $4.2\;\times \;10^{-7}$), providing evidence of structural homology to a VP39-like methyltransferase domain. The coordinates and fragment assignments of these predictions are summarized in Table 5.

### 3.4. Protein Homology Analysis

#### 3.4.1. HHpred Analysis

HHpred analysis of the translated recovered fragments identified no convincing remote-homology matches for Fragment_1 or Fragment_2. Fragment_2 showed only a weak match to a Beige/BEACH domain (PF02138; probability = 20.47%; E-value = $1.6\;\times \;10^{2}$), which was not considered significant. Fragment_3 yielded a moderate-probability match to the proteasome activator PA28/11S regulator (PF02252; PDB: 1AVO_J; probability = 75.65%), but the high E-value (19) indicated that this match was not statistically significant and it was therefore not interpreted further.

In contrast, a region within Fragment_3 showed multiple high-confidence HHpred matches to methyltransferase proteins, with probabilities ≥99.8% and E-values between $7.2\;\times \;10^{-22}$ and $2.3\;\times \;10^{-20}$. The matched templates included tRNA methyltransferases, cap-specific mRNA methyltransferases, an African swine fever virus methyltransferase, and a nsp2 protease/methyltransferase. The concordance of these independent high-confidence matches supports the presence of a methyltransferase-like domain within Fragment_3. The five highest-ranking HHpred matches are summarized in Table 6.

#### 3.4.2. HHblits Analysis

HHblits analysis identified strong homology to the TcTV8 polyprotein for Fragment_1 and Fragment_3, whereas no convincing homologous matches were obtained for Fragment_2. Fragment_1 showed a highly significant match to the TcTV8 polyprotein (probability = 100%; E = $8.8\;\times \;10^{-150}$). The remaining matches for Fragment_1 had substantially higher E-values (6.4 and 8.1) and were not considered significant.

For Fragment_2, the three highest-ranking matches were to uncharacterized or hypothetical proteins and to an FkbM methyltransferase, with probabilities ranging from 85.2% to 88.0%. However, all corresponding E-values were >1 (1.7–3.2), providing insufficient evidence for confident homology or functional assignment. Therefore, the FkbM methyltransferase match was not interpreted as evidence of methyltransferase function.

Fragment_3 showed a 100% HHblits probability match to the TcTV8 polyprotein (A0A0P0QKN0; E-value = 1.2 × 10^−316^), supporting homology between this recovered fragment and the TcTV8 polyprotein. The lower-ranking matches to an uncharacterized protein and a SIS-domain protein had E-values of 2.1 and 4.2, respectively, and were not considered statistically significant. The three highest-ranking HHblits matches for each recovered fragment are summarized in Table 7.

### 3.5. Sequence Similarity to TcTV8

BLAST analyses consistently demonstrated that the three recovered fragments are likely derived from TcTV8 and correspond to regions of its large polyprotein. For Fragment_1, BLASTP revealed a significant match to the TcTV8 polyprotein (YP_009179217.1), aligning over 81 aa with 100% query coverage and 100% amino-acid identity (E = 6 × 10^−46^). At the nucleotide level, BLASTN identified a 246 nt fragment mapping to the TcTV8 TCP-2 polyprotein gene (NC_028367.1) with 100% query coverage, 89.39% nucleotide identity, and a highly significant E-value (0.0). This assignment was further supported by BLASTX, which detected the TcTV8 polyprotein (YP_009179217.1) with 100% query coverage, 87.00% amino-acid identity, and a highly significant E-value (0.0). In contrast, Fragment_2 and Fragment_3 showed robust and concordant matches across all three BLAST modalities. BLASTP revealed that both sequences correspond to the TcTV8 polyprotein, with Fragment_2 aligning over 168 aa (100% coverage, 94.05% identity, E = 2 × 10^−103^) and Fragment_3 over 644 aa (100% coverage, 93.17% identity, E = 0.0), indicating that Fragment_3 represents a much larger portion of the viral polyprotein. These results were mirrored at the nucleotide level, where Fragment_2 (504 nt; 83.33% identity, E = 2 × 10^−130^) and Fragment_3 (1932 nt; 86.70% identity, E = 7 × 10^−131^) both matched the TcTV8 TCP-2 polyprotein gene (NC_028367.1) with full query coverage. BLASTX further confirmed these assignments, with Fragment_2 (504 nt, 100% query coverage, 100.00% identity, E = 1 × 10^−91^) and Fragment_3 (644 aa, 99% query coverage, 94.05% identity, E = 9 × 10^−92^) mapping to the TcTV8 polyprotein (YP_009179217.1). Collectively, these data indicate that all three sequences derive from Tacheng tick virus 8, with Fragment_1 representing a short coding region and Fragment_2 and Fragment_3 corresponding to progressively larger segments of the viral polyprotein. A summarized table of the BLAST results is provided in Table 8.

### 3.6. Phylogenetic Placement of the Recovered TcTV8-like Sequence

**Figure 2:**
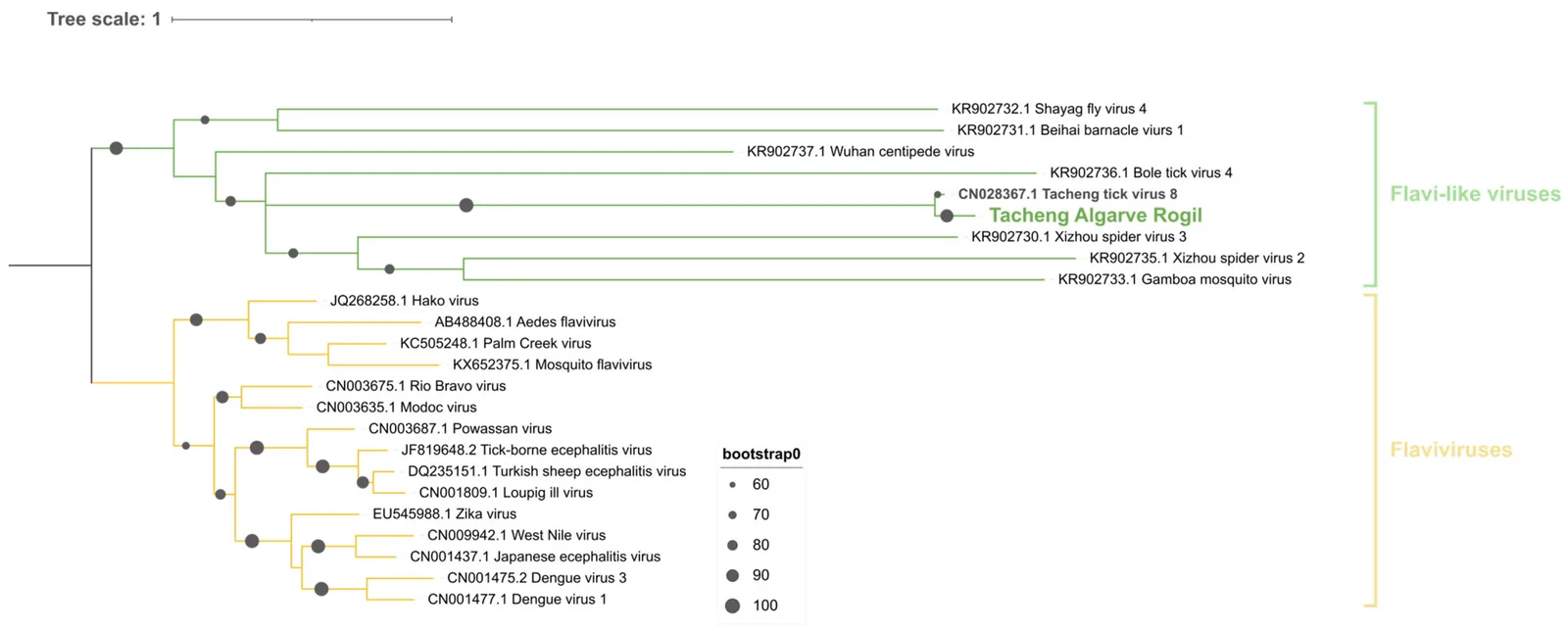
Maximum-likelihood phylogenetic placement of the recovered Portuguese TcTV8-like sequence among TcTV8 and related flavi-like viruses, based on a nucleotide alignment of homologous regions shared between the recovered sequence and the reference taxa. The tree was inferred in IQ-Tree using the TIM3 + F + R3 substitution model, selected by ModelFinder according to the Bayesian Information Criterion (BIC). Node support was assessed with 1000 ultrafast bootstrap replicates and is represented by proportional circles at the corresponding nodes. The Portuguese sequence (“Tacheng Algarve Rogil”) and the TcTV8 reference sequence (RefSeq NC_028367.1) are highlighted within the flavi-like virus lineage. The tree was rooted at the midpoint.

Maximum-likelihood phylogenetic analysis placed the recovered Portuguese TcTV8-like sequence close to a previously reported sequence from Tacheng, China. The Portuguese sequence grouped within the TcTV8-like lineage, with the corresponding node receiving 100% bootstrap support (Figure 2). Because the analysis was based on partial nucleotide sequence data from divergent viruses, deeper relationships within the broader flavi-like virus group were interpreted cautiously.

## 4. Discussion

This study provides preliminary evidence for the presence of Tacheng tick virus 8 (TcTV8)-like sequences in ticks collected in Portugal. To our knowledge, this represents the first report of TcTV8-like sequence data from the European Union, extending the documented geographic occurrence of this poorly characterized virus beyond previously reported detections in Asia and Anatolia [9,12,13]. However, this conclusion should be interpreted cautiously because the evidence derives from a single positive sample, a small number of viral reads, and three partial coding fragments rather than recovery of a complete viral genome.

Only one of the ten analyzed samples produced a coherent viral signal, with all 26 virus-classified reads assigned by Kraken2 to TcTV8. However, this taxonomic concordance should be interpreted cautiously given the small number of viral reads and the limited representation of closely related TcTV8-like viruses in reference databases. The absence of comparable TcTV8-like signals in the remaining samples suggests that the virus was not commonly detected in this small dataset, although low-abundance infections could have remained below the sensitivity of the sequencing approach. Conversely, because no negative extraction or library-preparation controls were included, low-level reagent contamination or barcode misassignment between multiplexed samples cannot be completely excluded. Detection of low-abundance RNA viruses in arthropod metagenomic datasets can be strongly influenced by sequencing depth, host-background RNA and amplification bias, particularly when sequence-independent amplification approaches such as SISPA are used [2,6,12,25,35].

Sequence- and profile-based analyses provided complementary support for the TcTV8-like assignment. Fragment_1 showed strong HHblits homology to the TcTV8 polyprotein, whereas Fragment_2 did not produce statistically convincing profile-based matches and therefore cannot presently be assigned a specific function with confidence. Fragment_3 was the most informative recovered region. InterProScan identified a VP39-like domain/superfamily match, while HHpred detected multiple high-confidence matches to structurally characterized methyltransferases. The convergence of these independent analyses supports the presence of a methyltransferase-like region within Fragment_3.

Methyltransferases are common components of the replication machinery of many positive-sense RNA viruses and, in canonical flaviviruses, participate in modification of the viral RNA cap through N7- and 2′-O-methylation [36,37,38]. These reactions contribute to efficient viral RNA translation and can also influence recognition of viral RNA by host innate immune mechanisms [39,40]. Nevertheless, the recovered TcTV8-like sequence is highly incomplete, and TcTV8 itself is evolutionarily distant from classical orthoflaviviruses. Accordingly, no inference regarding pathogenic potential can be drawn from this phylogenetic relationship, and no pathogenic role has currently been established for TcTV8. Therefore, the present data do not establish that the Fragment_3 methyltransferase-like region performs the same biochemical functions as the canonical flavivirus NS5 methyltransferase. Furthermore, the HHpred hits include proteins with different substrate specificities, including tRNA- and mRNA-associated methyltransferases. The results should therefore be interpreted primarily as evidence of structural and evolutionary homology to a methyltransferase-like domain rather than evidence of a specific enzymatic activity. This interpretation is consistent with previous observations that highly divergent flavi-like viruses can retain conserved replication-associated protein architectures despite substantial primary-sequence divergence [41,42]. 

Notably, no RNA-dependent RNA polymerase (RdRp) region was recovered from the Portuguese sample, despite screening of the recovered sequences. Because RdRp is one of the most conserved and phylogenetically informative regions in RNA viruses [43,44], its absence limits the resolution of the taxonomic assignment. The classification of the detected virus as TcTV8-like therefore relies on the combined evidence from taxonomic read assignment, sequence similarity to TcTV8 reference sequences, the methyltransferase-like region identified in Fragment_3, and phylogenetic placement of the available partial nucleotide sequence. Recovery of an RdRp-containing region or a more complete genome would enable more robust phylogenetic and taxonomic placement.

Phylogenetic analysis provided additional support for the TcTV8-like assignment by placing the Portuguese sequence close to the TcTV8 reference sequence within the broader flavi-like virus lineage. This is consistent with the sequence-similarity and profile-homology results. However, the phylogeny was inferred from partial nucleotide sequence data and included highly divergent taxa. Nucleotide substitution saturation and limited phylogenetic information in short alignments can reduce the reliability of deeper nodes, particularly across highly divergent RNA-virus lineages [45,46]. Consequently, the placement of the Portuguese sequence relative to TcTV8 is considerably more informative than the inferred relationships among the more distantly related flavi-like viruses.

The geographic significance of the Portuguese detection should likewise be interpreted cautiously. The finding is compatible with a wider distribution of TcTV8-like viruses than previously recognized, but a single positive sample cannot establish whether the virus is endemic in Portugal, has been recently introduced, or occurs more broadly in European tick populations. Arthropod-associated viruses are increasingly being identified through metagenomic surveys in regions where they had not previously been reported, and apparent geographic restriction can partly reflect differences in sampling intensity and sequencing effort [1,3,5,47]. Broader and systematic sampling will therefore be required before conclusions can be drawn about the distribution or dispersal history of TcTV8-like viruses [8,10,11].

An important limitation of this study is the restricted amount of viral sequence recovered. Although only 26 reads mapped to the TcTV8 reference genome, these reads collectively covered 30.18% of the reference at ≥1× depth. TcTV8 has a relatively large genome of approximately 19.5 kb, considerably longer than the ~10–12 kb genomes typical of classical flaviviruses [7,8,10,14]. The three recovered fragments totaled only 2682 bp, equivalent to approximately 13.7% of the 19,537 bp TcTV8 reference genome. Thus, breadth of read coverage should not be interpreted as equivalent to the proportion of the viral genome represented by the recovered sequences. Low read depth and discontinuous coverage substantially limited sequence recovery and functional characterization.

The combination of Oxford Nanopore sequencing and very low read depth may have contributed to uncertainty at poorly covered positions, particularly where consensus sequence reconstruction relied on very few reads. Although the accuracy of current Nanopore sequencing has improved substantially, consensus accuracy remains strongly dependent on read depth and appropriate polishing [48]. Independent confirmation of the recovered fragments by targeted RT-PCR followed by Sanger sequencing was not performed. Consequently, these sequences should be regarded as preliminary sequence-based evidence for a TcTV8-like virus rather than as a complete genome or definitive strain characterization.

The fragmented dataset also prevents conclusions regarding the biological properties of the detected virus. Detection of viral RNA in a tick does not by itself establish that the tick is a competent biological vector, because viral nucleic acid may originate from an actively replicating infection, from residual material associated with a recent blood meal, or from other environmental or biological sources [49]. Likewise, no conclusions can presently be drawn regarding vertebrate host range, pathogenicity or zoonotic potential. These distinctions are especially important for newly identified arthropod-associated viruses, many of which have no demonstrated association with disease [1,3,5,47].

Despite these limitations, the detection is relevant because the diversity and geographic distribution of TcTV8-like viruses remain poorly characterized. Metagenomic surveillance has considerably expanded the known diversity of RNA viruses associated with ticks and other arthropods, revealing numerous divergent lineages that would not readily be detected by targeted diagnostic assays [1,3,5,47]. The present finding supports further targeted surveillance of TcTV8-like viruses in Portugal and elsewhere in Europe. Future investigations should include RT-PCR confirmation of positive samples, targeted amplification of the recovered regions, deeper sequencing or viral-enrichment approaches, and recovery of complete or near-complete genomes. Expanded sampling across tick species, vertebrate hosts, seasons and geographic regions will also be necessary to determine the prevalence, genetic diversity and ecological associations of these viruses.

Overall, the results support the presence of TcTV8-like viral sequences in a Portuguese tick sample and provide partial molecular characterization of the detected virus. The strongest functional evidence was obtained from Fragment_3, which contains a methyltransferase-like region supported by complementary domain- and profile-based analyses. Together with its phylogenetic placement close to TcTV8, these observations support assignment of the recovered sequences as TcTV8-like. However, additional genomic and epidemiological data are required before conclusions can be drawn regarding the evolutionary history, host range, transmission biology or pathogenic potential of this virus.

## 5. Conclusions

This study provides preliminary evidence for the presence of Tacheng tick virus 8 (TcTV8)-like sequences in a tick sample collected in Portugal and, to our knowledge, represents the first report of TcTV8-like sequence data from the European Union. Three partial coding fragments were recovered, with Fragment_3 providing the strongest functional evidence through concordant domain- and profile-based homology to a methyltransferase-like region. Sequence-similarity analyses and phylogenetic placement further supported assignment of the recovered sequences as TcTV8-like. However, the limited number of viral reads, incomplete genome recovery, absence of an RdRp-containing region, and lack of independent molecular confirmation restrict the strength of the taxonomic and functional conclusions. No inference can presently be made regarding pathogenicity, host range, transmission competence, or zoonotic potential. These findings support further targeted surveillance and genomic characterization of TcTV8-like viruses in ticks from Portugal and other European regions.

## Figures and Tables

**Table 1:** Sample metadata for the ten ticks analyzed in this study, showing sample identifier, collection date and host species.

Sample No.	Collection Date	Host Species
1	November 2024	*Bos taurus*
2	October 2024	*Equus asinus*
3	July 2024	*Bos taurus*
4	October 2024	*Ovis aries*
5	November 2024	*Ovis aries*
6	November 2024	*Capra hircus*
7	October 2024	*Felix catus*
8	January 2025	*Bos taurus*
9	January 2025	*Corvus corone*
10	November 2024	*Capra hircus*

**Table 2:** Sample metadata for the ticks analyzed in this study, showing sample identifier and tick genus.

**Sample**	**Tick Genus**
1	Rhipicephalus
2	Rhipicephalus
3	Hyalomma
4	Dermacentor
5	Rhipicephalus
6	Rhipicephalus
7	Ixodes
8	Rhipicephalus
9	Unidentified
10	Rhipicephalus

**Table 3:** Read-length and quality statistics for the 54,797 Oxford Nanopore reads retained after quality filtering across all 10 barcoded samples, as reported by NanoPlot v1.43.0. Read counts and total bases meeting selected Q-score thresholds are also shown.

Metric	Value	Unit
Mean read length	978.5	bp
Mean read quality	16.5	Q-score
Median read length	794.0	bp
Median read quality	18.6	Q-score
Number of reads	54,797	total
Read length N50	1367.0	bp
STDEV read length	826.9	bp
Total bases	53,616,274	bp
**Q-score threshold ^1^**	**Reads**	**Total bases**
>Q10	54,661	53.6 Mb
>Q15	39,843	39.2 Mb
>Q20	22,722	21.8 Mb
>Q25	9182	7.5 Mb
>Q30	3324	1.8 Mb

^1^ Q-score thresholds indicate the number of reads and corresponding total bases with mean read quality at or above the specified Phred Q-score.

**Table 4:** Partial coding sequences corresponding to regions of the Tacheng tick virus 8 (TcTV8)-like polyprotein recovered from Sample 1. Start and end coordinates are given relative to the TcTV8 reference genome (KR902741/NC_028367) and refer to 1-based, inclusive positions on the reference sequence. All three sequences represent partial regions of the viral polyprotein and are incomplete at both ends.

Fragment	Start	End	Fragment Length (bp)	GC Content (%) ^1^	GenBank Accession
Fragment_1	12,621	12,866	246	47.15	PX975901.1
Fragment_2	13,663	14,169	504 ^2^	45.29	PX975902.1
Fragment_3	14,449	16,380	1932	42.90	PX975903.1

^1^ GC content was calculated excluding ambiguous nucleotide positions. ^2^ The 3-nucleotide difference observed relative to the reference sequence corresponds to a 3 bp deletion identified in our genome and does not reflect a sequencing or alignment error.

**Table 5:** Protein features and domain/superfamily matches identified by InterProScan v5.59-91.0 in the translated TcTV8-like coding fragments recovered in this study. For each prediction, the contributing InterProScan member database or method, associated identifier and description, coordinates of the matched region relative to the fragment, and E-value, where available, are shown.

Fragment	Database/Method	Identifier	Description	Start	End	E-Value
Fragment_3	Gene3D	-	Vaccinia virus VP39-like domain/superfamily	392	626	4.2 × 10^−7^

**Table 6:** Top HHpred matches obtained for the predicted methyltransferase region encoded by Fragment_3. Probability represents the estimated probability (%) that the query–template match reflects a true homologous relationship; E-value represents the expected number of false-positive matches with a score at least as good as the observed match; aligned columns indicate the number of matched columns in the query–template alignment.

Hit ID	Description	Probability (%)	E-Value	Aligned Columns
6JPL_B	tRNA methyltransferase	99.9	$7.2\;\times \;10^{-22}$	200
P0C969	African swine fever virus methyltransferase	99.9	$6.8\;\times \;10^{-22}$	202
4N49_A	Cap-specific mRNA methyltransferase 1	99.9	$2.4\;\times \;10^{-21}$	268
8P4E_O	Cap-specific mRNA methyltransferase	99.9	$2.0\;\times \;10^{-20}$	244
8DUF_A	nsp2 protease/methyltransferase	99.8	$2.3\;\times \;10^{-20}$	210

**Table 7:** Top three HHblits matches for the translated protein sequence encoded by each recovered TcTV8-like fragment against UniRef30 (MPI Bioinformatics Toolkit, accessed 20 December 2025). Probability represents the estimated probability (%) that the query–target match reflects true homology; E-value represents the expected number of false-positive matches with a score at least as good as the observed match. Hits are ranked by probability.

Query	Rank	Accession/ID	Description	Probability	E-Value
Fragment_1	1	A0A0P0QKN0	Polyprotein (Tacheng tick virus 8)	100.0	$8.8\;\times \;10^{-150}$
	2	A0A0Q8RTS3	DUF4136 domain-containing protein	81.28	6.4
	3	A0A2A5M638	Flagellin biosynthesis protein FlgL (Fragment)	79.61	8.1
Fragment_2	1	A0A419HTV4	Uncharacterized protein	88.0	1.7
	2	UPI0005961403	Hypothetical protein	85.7	3.0
	3	A0A813SE83	Methyltransferase FkbM	85.2	3.2
Fragment_3	1	A0A0P0QKN0	Polyprotein (Tacheng tick virus 8)	100.0	1.2 × 10^−316^
	2	A0A5J4X9C9	Uncharacterized protein	87.2	2.1
	3	A0A2V2D3B4	SIS domain protein	81.5	4.2

**Table 8:** BLAST results for the three recovered TcTV8-like fragments. For each analysis, the best-matching TcTV8 nucleotide or protein reference sequence is shown. Accession, accession number of the matched reference sequence; alignment length, number of aligned nucleotides for BLASTN or amino-acid residues for BLASTX/BLASTP; query coverage (%), percentage of the query sequence represented by the alignment; identity (%), percentage of identical positions across the alignment; E-value, expected number of database matches with a score at least as good as the observed match occurring by chance.

Fragment	BLAST	Best Hit	Accession	Alignment Length	Coverage (%)	Identity (%)	E-Value
Fragment_1	BLASTN	TcTV8 polyprotein gene	NC_028367.1	246 nt	100	89.39	0.0
	BLASTX	TcTV8 polyprotein	YP_009179217.1	246 nt	100	87.00	0.0
	BLASTP	TcTV8 polyprotein	YP_009179217.1	81 aa	100	100	6 × 10^−46^
Fragment_2	BLASTN	TcTV8 polyprotein gene	NC_028367.1	504 nt	100	83.33	2 × 10^−130^
	BLASTX	TcTV8 polyprotein	YP_009179217.1	504 nt	100%	100.00	1 × 10^−91^
	BLASTP	TcTV8 polyprotein	YP_009179217.1	168 aa	100	94.05	2 × 10^−103^
Fragment_3	BLASTN	TcTV8 polyprotein gene	NC_028367.1	1932 nt	100	86.70	7 × 10^−131^
	BLASTX	TcTV8 polyprotein	YP_009179217.1	644 aa	99	94.05	9 × 10^−92^
	BLASTP	TcTV8 polyprotein	YP_009179217.1	644 aa	100	93.17	0.0

## Data Availability

Sequence data was published in GenBank under accession numbers PX975901–PX975903. The data presented in this study are available on request from the corresponding author.
